# Case Report: Left main coronary artery spasm in monozygotic twins patients with vasospastic angina

**DOI:** 10.3389/fcvm.2025.1716917

**Published:** 2025-12-18

**Authors:** Yuwei Liao, Xiukun Liu, Xin Yi, Luhui Huang, Jinming Liu, Dongming Xie, Jiayuan Ling, Jiahe Xie

**Affiliations:** 1 Key Laboratory of Prevention and Treatment of Cardiovascular and Cerebrovascular Diseases, Department of Cardiology, Ministry of Education, First Affiliated Hospital of Gannan Medical University, Gannan Medical University, University Town, Ganzhou, China; 2 Jiangxi Branch Center of National Geriatric Disease Clinical Medical Research Center, Gannan Medical University, University Town, Ganzhou, Jiangxi, China

**Keywords:** coronary artery spasm, vasospastic angina, left main coronary artery, genetics, identical twins, case report

## Abstract

Coronary artery spasm (CAS), a known cause of myocardial ischemia that can present as variant angina, is primarily triggered by intraoperative catheter manipulation. Evidence for a genetic predisposition to coronary artery spasm is scarce. This report describes a case involving identical twins with no conventional cardiovascular risk factors who successively developed CAS. Their coronary angiograms showed spontaneous spasm in left main coronary artery, left circumflex branch artery and left anterior descending artery. This case provides clinical evidence for a genetic predisposition to CAS, highlighting the critical need for familial screening—particularly the proactive screening of the other twin when one monozygotic twin is diagnosed with vasospastic angina.

## Introduction

Coronary artery spasm (CAS) is characterized by abnormal, transient constriction of epicardial coronary arteries, which can lead to reversible total or subtotal occlusion of the vessel lumen and vasospastic angina (1). Although numerous environmental triggers and modifiable risk factors are well-documented, the fundamental mechanisms governing coronary artery spasm are still poorly understood. While genomic studies suggest a role for genetic susceptibility, robust direct clinical evidence to substantiate these findings remains limited (2). We report a rare case of monozygotic twins who both presented with coronary artery spasm localized to the left main coronary artery. This case provides clinical evidence supporting a genetic predisposition to this condition.

## Case presentation

**Figure 1:**
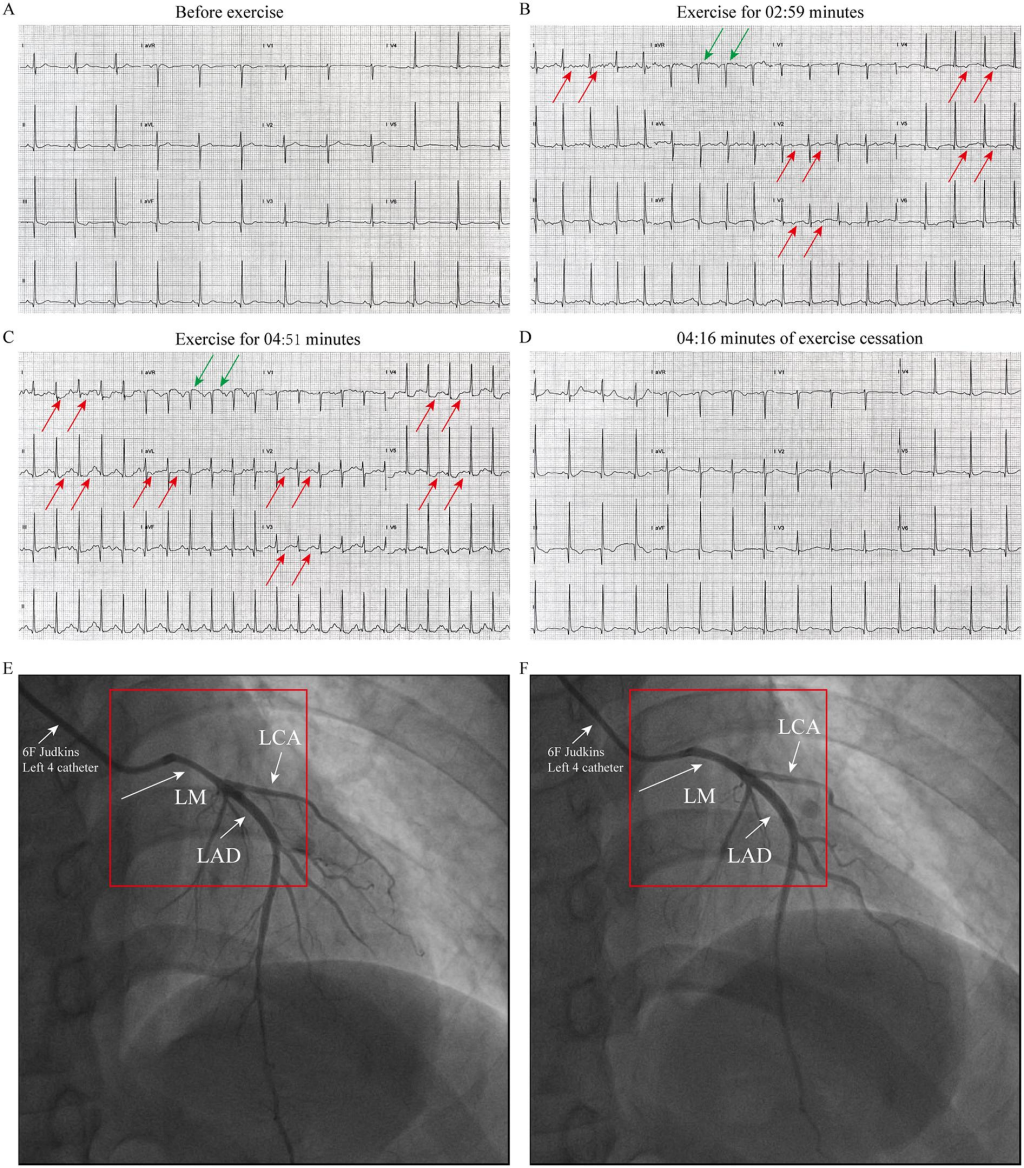
ECG and coronary angiography findings of case1: **(A)** baseline ECG (pre-exercise). **(B)** ECG at 02:59 min of exercise, demonstrating mild ST segment elevation in lead aVR and T-wave inversion and mild ST segment depression in lead I and V2–V5 (indicated by the arrow). **(C)** ECG at 04:51 min of exercise, showing ST segment elevation in lead aVR and persistent ST-segment depression in lead I, II, aVL, and V2–V5 (indicated by the arrow). **(D)** At 04:16 min cessation of exercise, ECG abnormalities resolved. **(E)** Coronary angiography performed with a 6F catheter reveals about 60% stenosis in the left main coronary artery (LMCA), left circumflex artery (LCX), and left anterior descending artery (LAD), as indicated by the arrow. **(F)** After resolution of spasm with TIMI III flow after intracoronary nitroglycerin administration (Indicated by the arrow).

**Figure 2:**
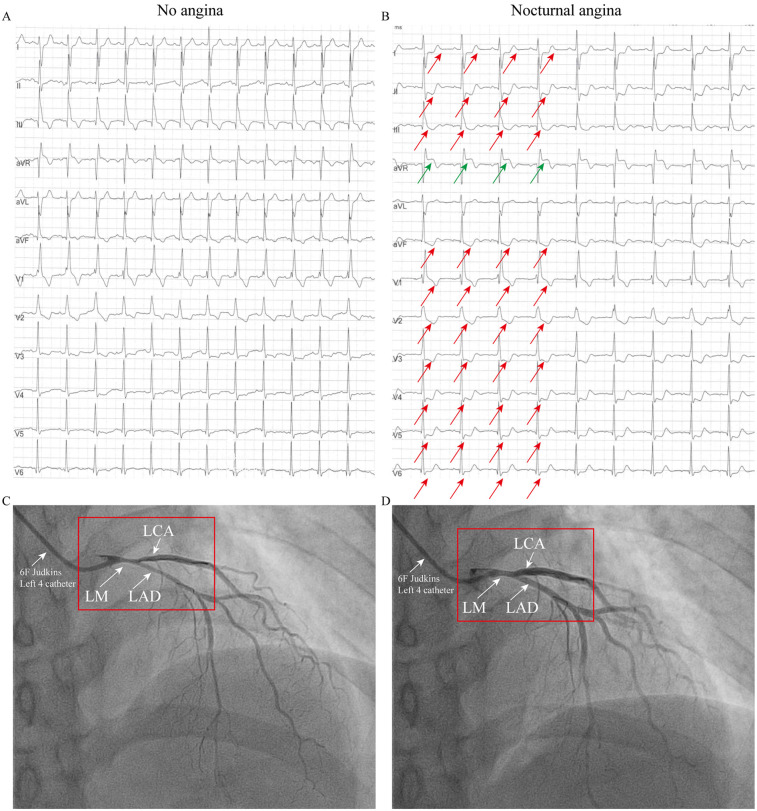
ECG and coronary angiography findings of case 2: **(A)** ECG during an asymptomatic period, showing a right bundle branch block (RBBB), T-wave inversions in leads III and aVF, and mild ST-segment depression in leads V3–V4. **(B)** ECG during chest pain showing acute ischemic changes: ST-elevation in aVR with diffuse ST-depression (I, II, V3–V6) and T-wave pseudonormalization in lead III (indicated by the arrow). **(C)** When the 6F catheter was inserted, about 60% spasm was observed in the left main coronary artery, the left circumflex artery and the left anterior descending artery (indicated by arrows). **(D)** Administration of 200 µg intracoronary nitroglycerin relieved the vasospasm (indicated by the arrow).

Patient 1 (Younger Sister): A 39-year-old woman was admitted in March 2025 for evaluation of recurrent episodes of chest pain. Each episode lasted approximately 10 min and resolved spontaneously. Her symptoms had intensified in the week preceding admission. She had no conventional cardiovascular risk factors for coronary artery disease (Supplementary Table 1). Vital signs and physical examination were unremarkable. Laboratory evaluations—including complete blood count, cardiac enzymes, troponin, liver and renal function tests, and lipid profile—were all within normal limits. Upon admission, the patient underwent a treadmill exercise test. The electrocardiogram (ECG) before exercise was nearly normal (Figure 1A). During the treadmill exercise test, the following findings were observed: After 02:59 min of exercise, ECG only showed mild ST-segment elevation in lead aVR and T-wave inversion and mild ST-segment depression in lead I and V2-V5(Figure 1B). By the 04:51-min mark, the patient developed chest pain, along with ST-segment elevation in lead aVR and persistent ST-segment depression in lead I, II, aVL, and V2–V5 (Figure 2C). All symptoms and ECG abnormalities resolved 04:16 min after cessation of exercise (Figure 2D). Coronary angiography demonstrated approximately 60% spasm in left main coronary artery, left circumflex branch artery and left anterior descending artery (Figure 1E) and a normal right coronary artery. The administration of 200 µg intracoronary nitroglycerine resulted in prompt relief of the spasm, with restoration of normal luminal diameter and coronary flow (Figure 1F). Her symptoms resolved completely under calcium channel blocker therapy, and she was subsequently discharged in stable condition.

Patient 2 (Older Sister): The patient's identical twin sister had been treated at another institution in 2023 for a three-year history of chest pain, occurring predominantly at night and often persisting for 10 min. She also had no conventional risk factors for coronary artery disease (Supplementary Table 1). When the patient was not experiencing chest pain, the ECG was essentially normal, showing a right bundle branch block (RBBB), T-wave inversion in lead III, aVF and mild ST-segment depression in lead V3–V4 (Figure 2A). During symptomatic episodes, ambulatory ECG monitoring captured significant ST-segment elevation in lead aVR, and marked ST depression in lead I, II, V3–V6 and T-wave pseudonormalization in lead III during symptomatic episodes (Figure 2B). Coronary angiography demonstrated approximately 60% spasm in left main coronary artery, left circumflex branch artery and left anterior descending artery (Figure 2C) and a normal right coronary artery. Administration of 200 µg intracoronary nitroglycerin successfully relieved the vasospasm (Figure 2D). She was likewise managed effectively with calcium channel blockers and discharged symptom-free.

## Discussion

CAS is a significant etiology of chest pain and plays a critical role in the pathogenesis of variant angina (Prinzmetal's angina) and acute coronary syndromes (ACS). We report a case of twin sisters with no traditional risk factors who experienced frequent episodes of severe rest angina. Electrocardiogram during chest pain showed significant ischemic changes, and coronary angiography demonstrated spasm of the left main coronary artery.

CAS is characterized by transient, intense constriction of an epicardial coronary artery, leading to a temporary reduction or cessation of blood flow. This phenomenon can occur in both angiographically normal and diseased vessels. CAS usually does not cause serious problems, but it can sometimes lead to a severe heart attack (myocardial infarction) (3). CAS is linked to heightened coronary artery reactivity. This hyperreactive state can be triggered by various factors, such as alcohol, magnesium deficiency, smoking, hyperlipidemia, chemotherapy, and chronic anxiety (2). While CAS is frequently catheter-induced during procedures, this etiology was unlikely in the present cases. The following evidence supports this conclusion: both patients had a history of recurrent chest pain accompanied by transient ST-segment changes on ECG, and their symptoms resolved after treatment with calcium channel blockers following discharge.

Epidemiological studies have indicated that the prevalence of CAS may be influenced by genetic factors. For instance, severe familial CAS has been reported in two siblings (4). A case-control genome-wide association study from the Biobank Japan suggested that variants in the RNF213 gene may contribute to CAS susceptibility (5). Additionally, the aldehyde dehydrogenase 2 (ALDH22) variant genotype, which leads to deficient enzyme activity, has been associated with CAS in Japanese populations (6). Another significant genetic association involves the paraoxonase gene Gln192Arg (Q192R) polymorphism (7). In line with these findings, Takayuki et al. documented the occurrence of spasms in male twins, further supporting a genetic predisposition to CAS (8). The prevalence of CAS varies substantially across countries and racial groups, with a notably higher incidence reported in Japan and East Asia compared to Western populations (9). This disparity is reflected in distinct genetic and pathophysiological associations. In East Asian populations, particularly Japanese cohorts, CAS has been linked to the ALDH2*2 genotype and the eNOS Glu298Asp variant. The eNOS Glu298Asp variant diminishes nitric oxide production, thereby impairing a critical mechanism for vascular tone regulation (6, 10). In contrast, studies in European populations have connected CAS to elevated plasma endothelin-1 (ET-1) levels and related risk loci in the END1 gene (11). Gene-environment interactions also contribute to CAS. Research by Yosuke et al. indicates sex-specific susceptibility loci, with the p22 phox gene associated with CAS in men, and the stromelysin-1 and interleukin-6 genes in women (12). Additionally, genetic studies of coronary artery disease suggest that shared vascular structure and phenotypic concordance contribute to familial risk (13).

Our report underscores the crucial role of genetics in the pathogenesis of CAS. We recommend that clinicians actively elicit a detailed family history, especially in angina patients without conventional risk factors, as it significantly increases the index of suspicion for a genetic predisposition. This practice is essential for accurate diagnosis and helps prevent misdiagnosis.

## Conclusion

We report a case of twin sisters without traditional cardiovascular risk factors who presented with severe rest angina. The diagnosis of CAS was confirmed by electrocardiogram and coronary angiography. This case provides clinical evidence supporting a strong genetic predisposition to CAS. Therefore, we recommend obtaining a detailed family history, particularly in patients who lack conventional risk factors. Furthermore, if one monozygotic twin is diagnosed with vasospastic angina, the other should thus be proactively screened—thereby raising clinical suspicion, avoiding misdiagnosis, and securing a timely and accurate diagnosis.

## Data Availability

The original contributions presented in the study are included in the article/Supplementary Material, further inquiries can be directed to the corresponding author.
